# Use of health services one year before primary colorectal cancer

**DOI:** 10.1186/s12913-023-09298-7

**Published:** 2023-03-23

**Authors:** Elina Hermiö, Karri Seppä, Heidi Ryynänen, Elli Hirvonen, Liisa Pylkkänen, Jutta Järvelin, Nea Malila, Janne Pitkäniemi

**Affiliations:** 1 Unit of Health Sciences, Faculty of Social Sciences, Tampere University, Tampere, 33014 Finland; 2 Cancer Society of Finland, Unioninkatu 22, 00130 Helsinki, Finland; 3 University of Turku, 20014 Turun Yliopisto, Turku, Finland; 4 Finnish Institute for Health and Welfare, PL 30, 00271 Helsinki, Finland; 5 University of Helsinki, Yliopistonkatu 4, 00100 Helsinki, Finland

**Keywords:** Health services research, Primary health care, Hospitals, special, Colorectal neoplasms, Critical pathways, Health care utilization

## Abstract

**Background:**

Colorectal cancer (CRC) patient pathways focus typically on periods after confirmed diagnosis and only limited data are available on pathways prior to cancer diagnosis. The aim of the study was to describe the use of health services (HS) one year before diagnosis among CRC patients in Finland. We also studied the role of sex, age, stage, and university hospital district in relation to the use of HS during the pre-diagnostic phase. This information is expected to bring light on the question why CRC is often not found in its early stages.

**Methods:**

Incident CRC cases (*N* = 3115) concerning year 2015 were retrieved from the Finnish Cancer Registry and linked with data from the Finnish Institute for Health and Welfare on primary or specialised care outpatient visits or inpatient episodes over one year prior to CRC diagnosis. We modelled the average number of HS events per patient using Poisson regression model with log-link. Change points for monthly HS event rates and 95% CIs one year before diagnosis were evaluated using Poisson broken line regression models.

**Results:**

Around 10% of patients diagnosed in 2015 had no events prior to cancer leaving 2816 CRCs in the study. Of all pre-diagnostic events (*N* = 23268), 86% were outpatient events and 14% inpatient episodes. More than half of the inpatient episodes (65%) started as urgent admissions. The use of HS started to increase 3–4 months before diagnosis. The average number of pre-diagnostic HS events per patient varied by sex, age, stage and university hospital district. Overall, men had more events per patient than women and older patients had more events than younger patients.

**Conclusions:**

The amount of inpatient episodes starting as urgent admissions indicate potential bottlenecks in the access to health services. An increase in service use only 3–4 months prior to diagnosis reflects a need for advice both for health care professionals and the general population in recognising symptoms of CRC.

## Background

In Finland, on average 35 000 new cancer cases are diagnosed per year and approximately 13 000 die from cancer annually [1]. Colorectal cancer (CRC) is the second most common cancer among women and men in Finland with over 3400 new cancer cases and 1200 deaths per year. The five-year relative survival of CRC patients in 2016–2018 was 66% [2].

Cancer burden can be reduced by prevention, early detection, and improving cancer treatment [3, 4]. Known risk factors for CRC are overweight or obesity, heavy alcohol use, long term smoking and physical inactivity. Screening for CRC [5–7] together with early diagnostics [3] are seen as key elements when improving patient outcomes.

In Finland, population-based cancer screening programs for breast and cervical cancer are in place, and the patient pathways for these cancers are well defined with proper guidelines, registration and follow-up starting from invitation to screening and ending at death [7]. Population-based screening for CRC in voluntary municipalities among the 60–68-year old population was running in 2004–2016 in Finland [7–9]. However, only about 25% of the target population was invited in 2015 and about 70% attended (Malila personal communication). In all, there are limited data available about the use of health services (HS) among colorectal cancer patients before CRC diagnosis in Finland, and many of the cancers are diagnosed when already spread to regional lymph nodes or at metastatic stage [10]. In this context, understanding the cancer patient´s pathway before diagnosis is crucial.

Municipalities are responsible for the organization of health care services in Finland. Public HS include both primary and specialized health care, and these services are accessible to all citizens regardless of income, insurance or working status. For specialized care, one needs a referral from a primary care physician or a private practitioner. When there is a need for emergency visits, i.e., in case of an acute illness, the patient can be admitted to hospital without a referral. Every municipality belongs to a hospital district responsible for arranging specialized HS. The twenty hospital districts in Finland form five large catchment areas of specialized medical care with one university hospital in each area. Starting from the beginning of the 2023 there will be 21 wellbeing services counties which are responsible arranging health services. Due to long distances with scattered population in the northern and eastern regions, these university hospital districts differ in population size. Cancer treatment is provided by specialized care in Finland. However, cancer diagnostics from symptoms to first contacts with the HS typically starts in primary care [11].

Patient pathway is a complex concept [12] and there are several descriptions for it. Usually, patient pathways focus on periods after confirmed diagnosis (care or treatment phase). Recently, researchers especially in Europe, have shown increased interest in the pre-diagnostic phase and four studies focused on the use of primary care services before cancer diagnosis [13–16]. According to Ewing et al. [13], 87% of cancer patients had consulted a primary care doctor within 12 months before cancer diagnosis. Among adolescents and young adults, the use of primary care services increased several months before any cancer diagnosis [14]. Jensen et al. [15] discovered that among cancer patients general practitioners (GP) consultation frequency started to increase four to six months before cancer diagnosis and Ewing et al. [13] showed increased consultation frequency within 50–100 days prior to diagnosis. In addition Brandenbarg et al. [16] found that patients with CRC had more GP contacts compared with controls a year preceding cancer diagnosis.

Typically, there are no symptoms in CRC in its early stages [17]. Koskenvuo et al. [10] found that large proportion of the CRC patients in the Finnish randomized CRC screening programme in 2004–2011 were diagnosed when cancer was already metastasized. In addition, over 14% of CRC patients had an emergency surgery. These findings indicate there may be obstacles in the pathway of CRC patients from recognition of symptoms to definite diagnosis. As CRC should preferably be diagnosed in its early phase to improve patient outcomes, it is essential to study the use of HS and factors possibly causing delays in diagnosis.

The aim of the study was to describe the use of health services within one year before diagnosis among CRC patients in Finland. We also studied the role of sex, age, stage, and university hospital district in relation to the use of HS during the pre-diagnostic phase. This information is expected to bring light on the question why CRC is often not found in its early stages.

## Methods

Registers used in this study were the Care Register for Health Care (Hilmo), the Register of Primary Health Care visits (Avohilmo) and the Finnish Cancer Registry (FCR). Hilmo includes information on all inpatient episodes in primary and specialized care and outpatient visits in specialized care, including emergency care visits. Avohilmo includes information on all primary healthcare contacts, for example visits to GPs at health centres. All registers are controlled by the Finnish Institute for Health and Welfare (THL) and legally entitled to receive data regularly from public health care providers with high coverage [18, 19]. The data on outpatient visits and inpatient episodes are extracted from the patient record systems and then submitted to the THL national registries. The FCR includes information on all cancer cases in Finland since 1953 with detailed information on the patient, type of cancer (primary site, histology, date of diagnosis and method of confirmation) and date and cause of death. The health care professionals (including pathology laboratories) are obliged to report cancer cases to the FCR [20].

In this study primary health services refer to outpatient GP visits in primary care, whereas the specialized health services refer to outpatient visits made in specialized care and any inpatient episodes, both in specialized or primary care. An HS event refers here to any outpatient visit or any inpatient episode within primary or specialized health services including all diagnoses coded with ICD10 classification. An event is dated either at the date of any outpatient visit (primary or specialized care) or the start date of an inpatient episode within one year before CRC diagnosis.

From the FCR we obtained information on gender, date of birth, municipality, stage of cancer, age of patient at diagnosis of CRC and all incident colorectal cancers (ICD-10 codes C18-C20) [21] diagnosed in 2015. The dates for primary and specialized care outpatient visits and start and end date of inpatient episodes were extracted from Hilmo and Avohilmo databases. Then Avohilmo and Hilmo data were individually linked to the FCR data for one full year before diagnosis (2014–2015) using the patient´s personal identification code. The time-period of one year prior to diagnosis was selected based on studies showing increased use of HS within few months before diagnosis [13–15].

Age at one year before diagnosis was classified into five groups: < 50 years, 50–64 years, 65–74 years, 75–84 years, and 85 years and over. Stage of cancer was classified into three levels: localized (including localized and locally advanced T1-T4 + N0), non-localized (including regional lymph node and distant metastases), and unknown. Four patients had simultaneously multiple primary CRC tumours and we selected the one with the highest stage.

We included HS events that had occurred between 15 and 365 days before CRC diagnosis. This time period was chosen because the events in HS centred one year before diagnosis and because the cancer diagnoses were registered by one month accuracy in the FCR. In all, 23,268 pre-diagnostic HS events either in primary or specialized care were recorded in 2014–2015. HS events were categorized into five university hospital districts, Helsinki being the most urban, and Oulu the most rural; in others there are both urban and rural areas, according to the patient’s municipality of residence. Of all HS events 38% were in Helsinki university hospital district, following Tampere (20%), Turku (18%), Kuopio (17%), and Oulu (8%).

We report the average number of pre-diagnostic HS events per incident CRC patient per year as primary outcome. The average number of HS events per patient was modelled using Poisson regression model with log-link and we explored association between four factors (sex, age, stage and university hospital district). Main effects and all interactions between these four factors were evaluated using the likelihood ratio test when comparing nested models. We report numbers of patients and age-adjusted average numbers by sex, stage, and university hospital district over one full year period prior to cancer diagnosis. The age-adjusted numbers and their 95% confidence intervals were estimated using Bayesian Poisson regression model and weights based on the total number of patients in the five above-described age categories. Monthly HS event rates were estimated by dividing the number of all events by person time in a given month. We report monthly change points for HS events rates and 95% CIs one year before diagnosis using Poisson broken line regression models stratified by sex, primary and specialized care [22, 23].

All analyses were done using R and regression models with broken line using R package segmented (version 4.0.0).

## Results

We identified in all 3115 patients diagnosed with CRC in 2015, 1452 (47%) women and 1663 (53%) men. Median age of the patients was 71 years (range 22–96) among men and 73 years (range 19–103) among women. Of all patients, 299 (10%) had no HS events within the year preceding diagnosis and our analyses included 2816 patients (90%) with at least one HS event within a year before diagnosis. The proportion of male patients with no pre-diagnostic HS events was 11% and that of female patients was 8%. Among patients under 50 years of age, the proportion with no HS events was 18% in men and 17% in women, whereas only 4% of men and 5% of women aged 75–84 years had no pre-diagnostic HS events.

Among men, the highest proportion (37%) of pre-diagnostic HS events was in patients aged 65–74 years and among women, the highest proportion (30%) was in patients aged 75–84 years. The smallest proportion of pre-diagnostic HS events was found among the youngest (< 50 years) male and female patients (3% and 6%, respectively).

Of the total (*N* = 23268) pre-diagnostic HS events, 86% (*N* = 20010) were outpatient visits and 14% (*N* = 3258) were inpatient episodes within primary or specialized HS. More than half (65%) of the inpatient episodes started as urgent admissions (*N* = 2109). Of all the outpatient visits, 58% (*N* = 11603) were in specialized care and 6% (*N* = 698) of them were urgent visits. Of the primary care outpatient visits (*N* = 8407), 14% (*N* = 1158) were urgent. The proportion of men was slightly higher compared to women regarding urgent HS visits or hospital episodes.

In primary care, the outpatient HS event rate increased steeply 4.1 months (95% CI: 4.8 to 3.4) before diagnosis among men, and 3.2 months (95% CI: 3.8 to 2.6) among women. In specialized care, covering both outpatient visits and inpatient episodes, the trends were similar with rates increasing 2.4 (95% CI: 2.5 to 2.2) months before diagnosis among men and 1.4 (95% CI: 1.5 to 1.3) months among women.

The distribution of cancer stage was similar in both sexes (Table 1). Both in men and in women, the proportion of non-localized stage patients was highest, 45% of male and 43% of female patients. The proportion of localized stage cancer was roughly one third, and the stage was unknown in one fifth of patients. When studied by university hospital district, the highest proportion of male patients with localized cancer was in Helsinki district and among female patients in Kuopio district. On the other hand, the highest proportion of male patients with non-localized cancer was in Oulu district and among female patients in Helsinki district Table 1.

**Table 1:** Number and proportion of incident CRC patients by stage, sex and university hospital district in 2015

Sex		Helsinki (*N* = 1040)	Turku (*N* = 580)	Tampere (*N* = 649)	Kuopio (*N* = 518)	Oulu (*N* = 328)	**Total (*N* = 3115)**
Men	Stage						
	Unknown	81 (14.7%)	74 (22.9%)	87 (25.7%)	63 (23.8%)	39 (21.0%)	344 (20.7%)
	Localized	219 (39.8%)	115 (35.6%)	99 (29.2%)	81 (30.6%)	54 (29.0%)	568 (34.2%)
	Non-localized	250 (45.5%)	134 (41.5%)	153 (45.1%)	121 (45.7%)	93 (50.0%)	751 (45.2%)
Women	Stage						
	Unknown	82 (16.7%)	74 (28.8%)	74 (23.9%)	60 (23.7%)	29 (20.4%)	319 (22.0%)
	Localized	184 (37.6%)	84 (32.7%)	92 (29.7%)	98 (38.7%)	49 (34.5%)	507 (34.9%)
	Non-localized	224 (45.7%)	99 (38.5%)	144 (46.5%)	95 (37.5%)	64 (45.1%)	626 (43.1%)

The average number of pre-diagnostic HS events per patient varied by sex (χ^2^ = 43.21,1 df., *p* = 4.89e-11) and age (χ^2^ = 313.81,4 df., *p* = 1.13e-66). The overall age-adjusted average number of pre-diagnostic HS events per patient was higher among men (8.6; 95% CI: 8.4–8.7) than among women (7.9; 95% CI: 7.8–8.1) (Table 2).

**Table 2:** Average number of pre-diagnostic HS events per CRC patient and standard deviation by sex, age groups and university hospital districts

	**University hospital district**					
**Age group**	Helsinki	Turku	Tampere	Kuopio	Oulu	**Total**
**Men**						
Under 50	5.9 (5.2)	4.6 (6.5)	7.6 (7.1)	4.9 (4.4)	3.8 (1.8)	**5.6 (5.2)**
50–64	8.1 (11.2)	6.3 (6.2)	6.3 (7.3)	6.3 (8.8)	6.1 (6.8)	**6.9 (8.7)**
65–74	10.1 (12.1)	6.6 (5.7)	9.2 (14.6)	11.0 (22.1)	6.2 (5.4)	**9.0 (13.6)**
75–84	10.7 (12.4)	10.8 (17.9)	8.0 (7.4)	8.5 (7.6)	7.3 (5.0)	**9.5 (11.9)**
Over 85	11.9 (22.3)	10.0 (7.7)	9.8 (8.8)	8.4 (7.0)	6.6 (5.4)	**10.0 (14.0)**
**Total**	9.6 (12.7)	8.2 (11.6)	8.3 (10.7)	8.7 (14.6)	6.4 (5.6)	**8.6 (11.9)**
**Women**						
Under 50	8.7 (10.8)	5.2 (3.3)	5.9 (4.4)	7.7 (11.1)	8.2 (5.9)	**7.4 (8.5)**
50–64	7.1 (7.0)	7.5 (10.0)	5.9 (5.3)	8.8 (11.8)	5.6 (7.1)	**7.1 (8.3)**
65–74	8.8 (8.3)	7.0 (5.8)	7.1 (8.3)	6.5 (5.4)	5.0 (4.0)	**7.4 (7.4)**
75–84	10.7 (13.0)	7.7 (5.7)	8.3 (6.5)	7.3 (6.8)	7.0 (5.6)	**8.5 (8.8)**
Over 85	10.2 (14.1)	8.5 (5.8)	7.6 (5.8)	10.0 (9.9)	8.5 (7.1)	**9.2 (10.0)**
**Total**	**9.0 (10.4)**	**7.5 (6.8)**	**7.2 (6.8)**	**7.9 (8.7)**	**6.4 (5.9)**	**7.9 (8.5)**

In males, the number of pre-diagnostic HS events per patient increased more rapidly by age than in females (χ^2^ = 69.99,4 df., p_interaction(sex,age)_ = 2.28e-14, Table 2). We found that the youngest (< 50 years) male patients had the lowest average number of HS events compared to other age groups and the number of pre-diagnostic HS events almost doubled with increasing age in men, while the number of HS events increased only slightly with increasing age in women (Table 2).

**Fig. 1:**
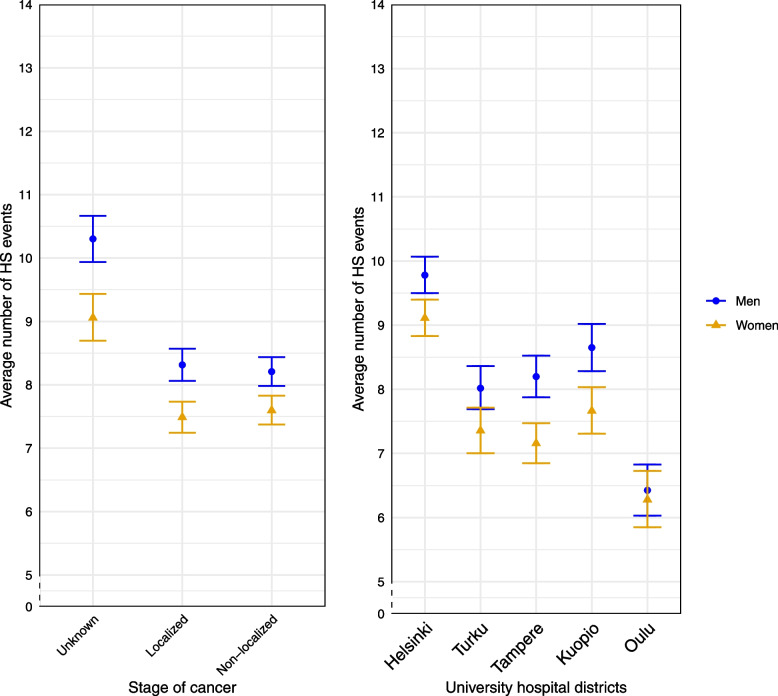
Age-adjusted average number of pre-diagnostic HS events per CRC patient with 95% confidence intervals by sex and stage of cancer (left panel) and age-adjusted average number of pre-diagnostic HS events per CRC patient with 95% confidence intervals by sex and university hospital districts (right panel)

The average number of pre-diagnostic HS events varied differently by age and stage (χ2 = 157.89, df = 8, p for interaction (age, stage) = 4.42e-30), but not by sex and stage (χ2 = 7.67, df = 2, p for interaction (sex, stage) = 0.021). The age-adjusted average number of pre-diagnostic HS events by sex and stage of cancer are shown in Fig. 1, left panel. In the right panel we illustrate the age-adjusted average number of pre-diagnostic HS events by sex and university hospital district. In men the age-adjusted average number of pre-diagnostic HS events was higher than in women in all stages. The average number of age-adjusted pre-diagnostic HS events was significantly higher in unknown stage of cancer compared to localized and non-localized stage of cancer in both sexes. (Fig. 1, left panel.)

The highest age-adjusted average number of pre-diagnostic HS events per patient was seen in Helsinki university hospital district and the lowest in Oulu university hospital district. In every university hospital district, except in Oulu, the average age-adjusted number of HS events was higher in men than in women, i.e. the number of HS events by university hospital districts was not modified by sex (χ2 = 5.40, df = 4, p for interaction (sex,univ.hosp.) = 0.25, Fig. 1, right panel.)

**Fig. 2:**
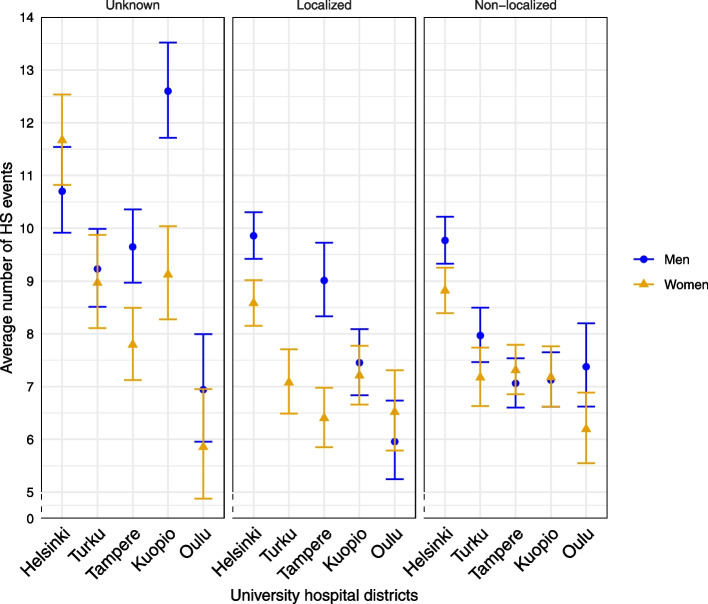
Age-adjusted average number of pre-diagnostic HS events per CRC patient by sex, stage of cancer and university hospital districts with 95% confidence intervals *Footnote: The age-adjusted estimate of male patients with localized cancer in Turku university hospital district was not available, because there were no patients in the youngest age category

The average number of pre-diagnostic HS events varied differently between sex, university hospital district and stage (χ^2^ = 51.16, df = 8, p for interaction (sex, univ.hosp, stage) = 2.45e-8, Fig. 2). There was a difference between men and women in the average number of pre-diagnostic HS events in localized stage of cancer in Helsinki, Turku and Tampere university hospital districts, but only in Helsinki university hospital district in non-localized stage of cancer. Among cancers of unknown stage, the differences between university hospital districts (in age-adjusted average number of pre-diagnostic HS events) were highest (Fig. 2).

## Discussion

We studied the use of HS among CRC patients within one year before cancer diagnosis in Finland. The results showed that the closer the CRC diagnosis was, the more health services were used. We found also that the monthly HS event rate started to increase earlier in primary care than in specialized care. In men the HS event rate started to increase earlier than among women in primary and specialized care. Our results are in line with those observed in earlier studies [13–15] showing that an increase in HS events is mainly seen within a few months before diagnosis, although the health care systems in Finland, Denmark and Sweden are slightly different. For example, there are standardized cancer patient pathways in Denmark and Sweden [24, 25] which are not as such available in Finland. These cancer patient pathways give the GPs an opportunity to urgently refer patients with alarming symptoms potentially related to cancer to a standardized diagnostic pathway. Our results also support the decision to use the one-year time-period before diagnosis to sufficiently describe the use of health services among colorectal cancer patients before the diagnosis is finally made.

Overall, the average number of pre-diagnostic HS events per patient was slightly higher among men compared to women. This finding differs from previous studies [26, 27], where women have been observed to use HS more than men. Also, in Finland in 2018, 55% of all primary care outpatient events were among women and 45% among men [28]. In contrast, we found that men with undiagnosed CRC used more HS than women. This could be related to differences in the anatomical site of CRC and symptoms related to colon cancer being less clear compared to symptoms of rectum cancer [18, 29]. In Finland, CRC in women is more often proximal than in men: the age-adjusted incidence of colon cancer being 42/10^5^ in men and 34/10^5^ in women, and that of rectum cancer 29/10^5^ and 16/10^5^, respectively [30]. In women, the proportion of colon cancers out of all colorectal cancers was 68% in 2015 whereas this proportion was 59% in men.

We found that the younger the patients were, the lower the average number of HS events per patient was. This could be explained by the fact that older patients had more chronic diseases and therefore used HS more often. Another possible explanation might be that young patients used primarily private occupational health care as a first contact in which case the HS events were not captured in this study. In addition, in men the average number of pre-diagnostic HS events per patient started to increase at younger age than in women. However, women in the youngest age group had more HS events per patient than men, which may be related to more frequent reproductive health related visits among women including e.g., participation in breast and cervical cancer screenings.

The proportion of patients with no HS events before diagnosis was surprisingly high, 10% of patients diagnosed in 2015. The distribution of cancer stage was similar among men without pre-diagnostic events in HS compared to men with pre-diagnostic events. However, among women with no pre-diagnostic events in HS, the distribution of cancer stage was different compared to women with pre-diagnostic events in HS. The proportion of women with no pre-diagnostic events in HS was higher in women with non-localized cancer or cancer with unknown localization at diagnosis than that of women with localized cancer.

We found that 65% of the inpatient episodes started as urgent admissions, meaning that the patient was diagnosed with cancer at emergency surgery. This finding may indicate delays or obstacles in the patient pathway. Based on previous studies [10, 17] some patients inevitably end up with emergency surgery. However, avoiding emergency surgery is expected to improve the quality of surgery and hence improve CRC outcomes, including survival [31, 32].

Our study showed differences between university hospital districts in the average number of pre-diagnostic HS events per patient. This finding may be related to different clinical practices or organizational guidelines on managing patients when suspecting cancer. Identifying cancer in primary care can be challenging also because of the unspecific symptoms, lack of clinical expertise or lack of physicians in certain areas. Moreover, it might be that patients with CRC do not to seek HS, because they don’t have symptoms, or they do not recognize CRC related symptoms as they may be similar to other common conditions. It is also possible that low threshold and better access to health services, i.e., short waiting times to primary care and possibility to consult specialists (e.g., in occupational health or other private provider), could explain the differences between university hospital districts. There are also regional differences in use of public and private sector HS, and most of the private sector HS providers are in southern Finland [11].

Some of the differences found between university hospital districts could be explained by environmental or socio-economic status related issues. For example, the Helsinki university hospital district is clearly the biggest one in size of population and the environment of the district is urban. On the other hand, age adjusted incidence rates of CRC varied between university hospital districts, Turku region having the highest incidence, following Helsinki, Tampere, Kuopio and Oulu among men and women [30]. There are also more HS providers in urban regions. In contrast, Oulu university hospital district has the lowest population and more rural areas with long distances to HS. Therefore, differences in the average number of HS events per patient could partly be a question of supply and demand.

Moreover, even though variation in survival of CRC patients between university hospital districts in Finland has decreased almost by 20% from 1997–2006 to 2007–2016, there are still regional differences [32]. Furthermore, CRC incidence was shown to be higher among those with high education and high socioeconomic status (SES) compared to those with lower education and low SES [33]. Differences were also found in CRC survival based on education [34] as patients with high education had higher survival than those with low education.

We found differences in pre-diagnostic health care utilization by sex, age, stage of cancer and university hospital district. The effect modification between sex, age, stage of cancer and university hospital district on the outcome was statistically significant. Also, all pairwise effect modifications except those between sex and stage or sex and hospital university district were statistically significant. Due to slow progression of colorectal cancer, stage at diagnosis is likely to reflect also the stage 12 months prior to diagnosis. However, because we have no information on the pre-diagnostic stage our results concerning association between use of HS and stage should be interpreted carefully.

Multiple datasets used in this study ensure the comprehensive coverage of the public Finnish health care system. Also, the registers used provide a solid base for this kind of study. The FCR data is overall accurate and close to complete for solid malignant tumours [19]. Coverage for colon cancer was 96% and for rectum cancer 97%. Also, validity and accuracy of the Hilmo data has been shown to be very good [18]. The Register of Primary Health Care visits is a relatively new register and therefore there are no validity studies available. However, the data concerning HS events are based on electronic health data submitted by HS providers, and the possibility for errors was rather small.

Because of the nature of register-based study, there are however some limitations. We have to accept that the data is as accurate as it is recorded and not all primary care providers could send their data comprehensively to THL [28]. In addition, even though registers were of high quality, there was a substantial number (one fifth) of CRC patients with unknown stage. This could be related to the lack of clinical expertise, poor clinical recording, or technical issues during extracting data.

Another potential limitation of this study was the fact that the HS events were considered between 15–365 days prior to a month-accurate diagnosis date. However, as we aim to describe use of HS before the CRC diagnosis, there is a high probability that the HS events occurring 0–14 days before cancer diagnosis were related to the diagnostic process of cancer.

## Conclusions

Our findings bring new light on the use of health services by CRC patients in Finland. They confirm the need for detailed information on patients’ journey from early symptoms to cancer diagnosis to solve the possible bottlenecks and thereby to reduce disease burden and cancer mortality.

The number of inpatient admissions starting as urgent admissions and an increase in service use 3–4 months prior to diagnosis indicate potential bottlenecks in recognizing symptoms related to CRC both among the health care professionals and the general population.

## Data Availability

The data that support the findings of this study are available from Finnish Cancer Registry (FCR) and Finnish Institute of health and welfare (THL) but restrictions apply to the availability of these data, which were used under license for the current study, and so are not publicly available. Data are however available from the authors (elina.hermio@tuni.fi, jutta.jarvelin@thl.fi) upon reasonable request and with permission of FCR and THL.

## Abbreviations

Avohilmo: Register of Primary Health Care Visits
CRC: Colorectal Cancer
FCR: Finnish Cancer Registry
GP: General Practioner
Hilmo: Care Register for Health Care
HS: Health Services
THL: Finnish Institute for Health and Welfare
